# Production of rumen- and gastrointestinal-resistant nanoparticles to deliver lysine to dairy cows

**DOI:** 10.1038/s41598-023-43865-6

**Published:** 2023-10-04

**Authors:** João Albuquerque, Ana R. Neves, Ingrid Van Dorpe, António J. M. Fonseca, Ana R. J. Cabrita, Salette Reis

**Affiliations:** 1 LAQV, REQUIMTE, Department of Chemical Sciences, FFUP, Rua Jorge Viterbo Ferreira n.º 228, 4050-313 Porto, Portugal; 2 School of Medicine and Biomedical Sciences (ICBAS), University of Porto, Rua Jorge Viterbo Ferreira n.º 228, 4050-313 Porto, Portugal; 3 CQM+-Centro de Química da Madeira, Universidade da Madeira, Campus da Penteada, 9020-105 Funchal, Portugal; 4 PREMIX-Especialidades Agrícolas e Pecuárias. Lda, Parque Indústrial II–Neiva, 4935-232 Viana do Castelo, Portugal; 5 LAQV, REQUIMTE, School of Medicine and Biomedical Sciences (ICBAS), University of Porto, Rua Jorge Viterbo Ferreira n.º 228, 4050-313 Porto, Portugal

**Keywords:** Biomaterials, Nanoparticles, Food nanotechnology, Zoology

## Abstract

Supplementing diets with rumen-protected lysine is a common strategy to meet the nutritional needs of high-producing dairy cows. This work addressed two separate but crucial issues: the lysine protection degree across the entire digestive tract as well as the production scalability of the proposed delivery systems. This was achieved by evaluating, in vitro or ex vivo, previously developed rumen-resistant lipid nanoparticles regarding their stability in the digestive tract and in the bloodstream of the dairy cow as well as how their production could be scaled-up. Results showed that the developed nanoparticles were able to resist digestion along the digestive tract but were degraded in the blood over 24 h. Thus, releasing their content to be used by the animal. In vitro viability assays were also performed, with the nanoparticles being found not to be inherently toxic when using nanoparticle concentrations up to 1 mg/mL. Results showed that neither the purity of the used lipids nor the production method significantly altered the nanoparticles’ properties or their ruminal resistance. Furthermore, the shelf-life of these nanoparticles was assessed, and they were found to retain their properties and remain usable after at least 1 month of storage. Moreover, a pilot-scale production allowed the production of nanoparticles with similar properties to the previous ones made using standard methods. To summarize, the proposed rumen-resistant nanoparticles presented potential as orally ingested lysine delivery systems for dairy cattle supplementation, being capable of a large-scale production using cheaper components while maintaining their properties and without any efficiency loss. It should however be noted that these results were obtained mainly in vitro and further in vivo bioavailability and production experiments are needed before this technology can be confirmed as a viable way of delivering lysine to dairy cows.

## Introduction

When considering amino acid (AA) supplementation in dairy cows, the rumen is considered the biggest obstacle due to the extensive microbial degradation that occurs in it^1,2^. To address this issue, several rumen protection technologies were developed, which aimed at producing delivery systems capable of protection AA from the ruminal microbiota^3–6^. However, adequate AA supplementation remains a dauting task in ruminants where limitations regarding poor protection and release issues^3,7^ are further complicated by difficulties in correctly measuring the true bioavailability of rumen-protected AA technologies^4^. The former usually leads to inadequate dietary formulations that, in turn, cause unbalanced AA levels in the plasma. Concomitantly, some studies have found that increased AA values in the blood are not consistently reflected in improved milk production^8,9^. The cumulative effect of these limitations renders improving the nitrogen-efficiency of dairy cows a harsh and complicated endeavor to this day, increasing both the costs and waste production of these animals^6,10^.

Moreover, rumen-protection systems should be designed to also take into account other organs that must be crossed after clearing the rumen: the omasum, the abomasum and the small intestine; where the effects of pH variations and enzyme activity should not be disregarded^5,11,12^. Most of the existing rumen-protection systems have been developed to be degraded, thus releasing their cargo, in one or across several of the former organs, which will ultimately be absorbed into the bloodstream at the intestine. However this can lead to reduced bioavailability mainly due to poor release^3,7,13^. The development of delivery systems that are themselves up-taken into the blood may reduce this loss, assuming that these systems are herein destroyed and their contents made available to the animal^10,14,15^, even if the degradation occurs after phagocytosis^16^. In a previous study, we have applied nanotechnology in the development of lysine (Lys) delivery systems for dairy cows, relying on solid lipid nanoparticles (SLN), composed of arachidic or stearic acids, to safely transport their cargo across the rumen^17^. However, and to ensure that these SLN can outperform the existing technologies, their stability in the remaining bovine digestive tract should also be assessed to better understand how they will supply Lys to the animal.

Additionally, for these technologies to reach the market, their production scale-up must be possible. Lipid nanoparticles, such as SLN, have been shown to be capable of scale-up^18,19^, with the added benefit of being regarded as effective and naturally biocompatible oral delivery systems in humans^20–22^. These SLN are most commonly produced by high-pressure homogenization (HPH), a method in which lipid emulsions are submitted to high pressures to form nano-emulsions that afterwards solidify into nanoparticle suspensions^23,24^. Another issue related with production scalability is the capability of resisting industrial production conditions, such as the high temperatures achieved during pelletization of animal feeds^25^.

In this work we evaluated the potential of SLN, already proven to resist ruminal digestion^17^, to deliver Lys into the bloodstream of dairy cattle. Therefore, these SLN were assessed, in vitro, for their ability to resist digestion in the remaining digestive tract and to degrade in the bloodstream, potentially releasing their Lys payload. The SLN’s in vitro toxicity was also evaluated and an HPH-based production method was proposed and compared to the standard sonication-based method used in the laboratory, also using cheaper components, to prove that the proposed SLN could go beyond the lab bench and reach the market.

## Results and discussion

### Nanoparticle stability in the abomasum

Incubation in abomasal conditions did not significantly alter the size of neither arachidic nor stearic acid SLN, for up to 1 h of incubation (Table 1), with only a tendency being observed in SLN composed of stearic acid, regarding a slight increase in size probably due to some adsorption of medium components. However, this increase is very small (less than 4% of the SLN size) and should, therefore, not be biologically relevant.

**Table 1:** Average size (nm) of solid lipid nanoparticles (SLN) composed of stearic or arachidic acids, without contact with the abomasal medium, with contact with the abomasal medium but where no incubation was allowed to occur (0 h of incubation) and after 1 h of incubation in the abomasal medium, n = 3.

	SLN			SEM	P value
	Stearic acid				
	No incubation	0 h	1 h		
Size (nm)	426	438	453	7	0.075

	Arachidic acid				
	No incubation	0 h	1 h		
Size (nm)	400	402	416	19	0.802

*SEM* standard error of the mean.

The obtained results show that both stearic and arachidic acid SLN should be capable of resisting digestion in the abomasum, at least for 1 h. This conclusion is also supported by the minimal digestion of lipids that occur in this organ^26^, and by the low susceptibility of the selected type of lipid SLN to pH variations^20,21,27^. Furthermore, stability of up to 1 h is adequate to ensure that the SLN can cross the abomasum, considering that there is an almost continuous flow of digesta in this organ. Indeed, the high feed intake of high producing ruminants^11^ render fast abomasal clearances^28,29^, with half-times as low as 13 min in cattle^30^. It should also be noted at this point, that a quantification of the Lys content of the SLN after this incubation was not possible so, even though the SLN were not degraded, the amount of Lys they carry could have been altered. However, since AA are not digested after the rumen, even if some Lys is released during this process, it should not affect its bioavailability as it would still be absorbed further down the digestive tract^5,11^.

### Nanoparticle stability in the small intestine

To ensure that all Lys that escapes the rumen reaches the blood, the SLN should, ideally, be themselves absorbed into the bloodstream and afterwards release their contents so it can be used by the animal. Studies have shown that particles with approximately 500 nm in size exhibit an increased intestinal uptake in humans^31^, however, analogous studies in bovines have not been reported. The proposed SLN present similar sizes and, for this reason, ensuring that they are not altered by the digestion processes that occur in the small intestine is important as they could translate to increased intestinal absorption of their Lys content.

In the small intestine, digestion takes place at near neutral pH and with the assistance of several enzymes. For this reason, and to create a more realistic model, the intestinal media was supplemented with a pancreatic enzyme complex, containing amylase, trypsin, lipase, ribonuclease and protease. The incubation of SLN in this complex intestinal-mimicking conditions did not significantly affect neither stearic nor arachidic acid SLN’s size (Table 2) after 2 h of incubation. This time period is routinely used in intestinal digestibility studies performed in cows^32^ and is also the indicated as the adequate time period in the use of an already existing in vitro duodenal model^33^.

**Table 2:** Size (nm) values of solid lipid nanoparticles (SLN) composed of stearic or arachidic acids, without contact with the intestinal medium, with contact with the intestinal medium but where no incubation was allowed to occur (0 h of incubation) and after 2 h of incubation in the intestinal medium n = 3.

	SLN			SEM	P value
	Stearic acid				
	No incubation	0 h	2 h		
Size (nm)	485	456	448	12	0.139

	Arachidic acid				
	No incubation	0 h	2 h		
Size (nm)	374	350	401	18	0.211

*SEM* standard error of the mean.

Considering that the proposed SLN are composed of saturated fatty acids, their resistance in the small intestine should not be compromised by the presence of lipases, since these enzymes mainly digest triglycerides. In fact, the natural resistance of these fatty acids to digestion, across the entire bovine digestive tract, was the reason for their selection as components for SLN production. The natural resistance of stearic and arachidic acid to lipase digestion, as well as these SLN’s natural resistance to pH variations^20,21,27^, explain why the incubation in intestinal conditions did not affect the integrity or dimensions of the SLN. It should be noted that a quantification of the Lys content of the SLN after this incubation was not possible, but as previously explained any potential Lys loss or release at this point should not influence its bioavailability^5,11^.

The presented results (Table 2) indicate that the proposed SLN retain their size after incubation in the intestinal medium, potentially enabling them to be absorbed into the bloodstream.

### Nanoparticle stability in the bloodstream

However, even if the SLN are absorbed at the small intestine, for Lys to be used by the dairy cow it must be released in the blood, hence becoming available^15,34^. To assess if the SLN could release their Lys content once in the bloodstream, an ex vivo stability assay was performed, using fresh bovine blood. Incubating the stearic acid SLN in this medium caused a significant reduction in their size after 3 h and rendered then statistically similar to the negative control after 24 h (Table 3). Regarding arachidic acid SLN, significant differences were found at all incubation timepoints except for 6 h, which remained similar to the initial SLN. Furthermore, the samples with SLN always showed particle sizes that were different when compared to the negative control (where no SLN were added) regardless of the timepoint, at least up to 24 h of incubation.

**Table 3:** Size (nm) values of solid lipid nanoparticles (SLN) composed of stearic or arachidic acids, without contact with blood, with contact with blood but where no incubation was allowed to occur (0 h of incubation) and after 3 h, 6 h, 9 h, 12 h and 24 h of incubation in blood, n = 3.

SLN		Size (nm)	SEM	P value
Control		6^a^*	3	
Stearic acid	Without contact with the blood	454^b^	38	< 0.001
	After 0 h of incubation	361^bc^		
	After 3 h of incubation	295^c^		
	After 6 h of incubation	293^c^		
	After 9 h of incubation	278^c^		
	After 12 h of incubation	275^c^		
	After 24 h of incubation	11^a^		
Arachidic acid	Without contact with the blood	401^b^	21	< 0.001
	After 0 h of incubation	402^b^		
	After 3 h of incubation	325^ce^		
	After 6 h of incubation	377^bef^		
	After 9 h of incubation	294^ cd^		
	After 12 h of incubation	326^cf^		
	After 24 h of incubation	243^d#^		

*SEM* standard error of the mean.

*This value is indicative of no present SLN (but of naturally occurring particles in suspension in the blood) as they were not added to the control.

^#^This value is not indicative of the total replicate populations, as in some of them no SLN were detected, indicating a possible destruction;

Different superscript letters indicate significant differences (P ≤ 0.05) between the negative control (blood without SLN addition) and each SLN at all timepoints. Stearic or arachidic acid SLN were compared only with the negative control and not with each other.

The differences in resistance between both SLN tested can be explained by the fact that the arachidic acid SLN have 25% more lipid in their matrix than stearic acid SLN, promoting a more rigid structure (due to a higher lipid/surfactant ratio) that is more difficult to degrade or simply take longer to degrade due to the higher amount of lipid present.

The slow degradation of SLN in the blood, particularly of stearic acid composed SLN, strengthens the hypothesis that, once in the blood stream, they would release their Lys content where it can be used by the animal. A slow degradation of the SLN would imply a release of Lys over time, which could benefit milk production, due to the mammary gland’s ability control AA influx (by regulating blood flow and specific AA extraction from the bloodstream) to better suit its current needs^35,36^. Since Lys is commonly found to be a limiting AA (particularly in corn-based diets), a steady and over-time supply of this AA, coupled with a selective mobilization of other, more abundant, components for milk synthesis by the mammary gland, would enable a continuous and increased production^14^. At the same time, Lys that remains within the SLN would remain protected from the natural occurring degradation and metabolism that AA suffer in the bloodstream or by some organs during circulation^14,34^. On the other hand, a burst release could lead to a temporary excess of Lys in the blood that wouldn’t be only used for milk production. This would cause an increase in Lys degradation or mobilization by other organs, reducing the overall Lys available to the mammary gland. The latter could lead to a reduction on the amount of Lys, supplied by the SLN, that would be used in milk component synthesis, thus reducing efficiency of the Lys delivery system. Further studies are required to validate this hypothesis, but the results presented indicate that SLN, particularly stearic acid SLN, were completely degraded in the blood after 24 h, which should release their Lys cargo directly into the bloodstream.

### Nanoparticle biosafety studies

**Figure 1:**
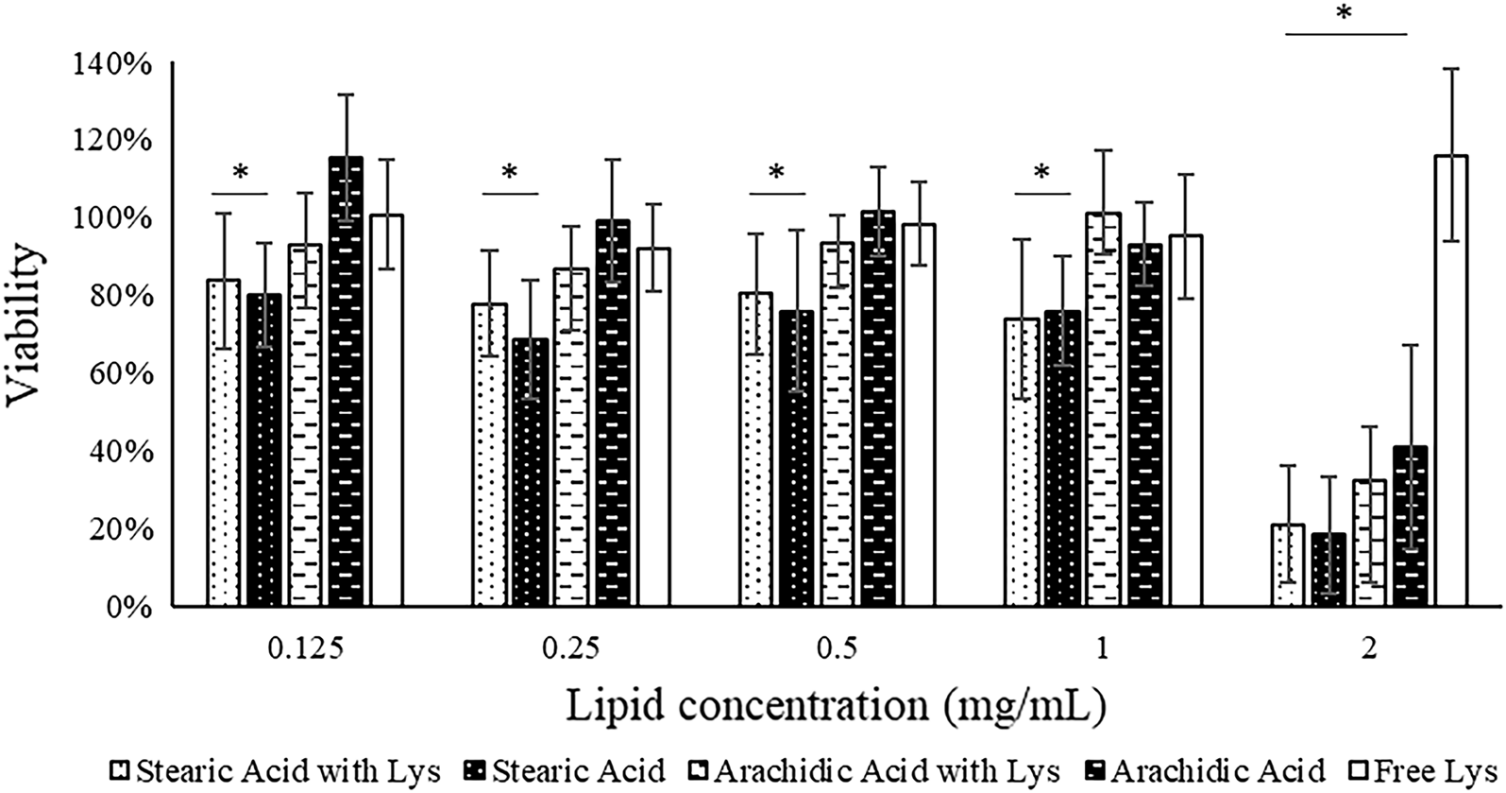
Cellular viability assay with L929 cells exposed to solid lipid nanoparticles (SLN) made of stearic or arachidic acids, with and without lysine (Lys), as well as free Lys, at increasing concentrations (0.125, 0.25, 0.5, 1 and 2 mg/mL of lipid). Free Lys concentrations were the same that were present in the respective Lys-loaded SLN. Values shown as mean ± standard deviation, n = 3. *Statistically different (P ≤ 0.05) from the positive controls.

Cellular studies are an important tool to provide preliminary data on biosafety, thus reducing the need to expose animals to several studies and tests. The MTT assay was used to determine the potential cytotoxicity of the developed SLN (Fig. 1), according to the existing regulations^37^. Free Lys showed no toxicity in all tested concentrations, while the SLN presented a significant cytotoxicity only at the highest concentration of 2 mg/mL. At lower concentrations, up to 1 mg/mL, the SLN presented high biocompatibility, reflecting approximately 80% and 100% of cellular viability, for stearic and arachidic acid, respectively.

All components used to produce the SLN are considered safe and possess high biocompatibility^20,38^. The results obtained not only confirmed the safety of the used SLN components, but also proved that their combination and structure in SLN form also did not present a high toxicity. Furthermore, if we consider the amount of Lys that RP-products are usually calculated to supply^6^, the animal would have to ingest a much higher dose-equivalent of SLN for toxic levels to be reached. The viability obtained for stearic acid SLN were found to be statistically lower than the control, but the difference between these values was not higher than 20%, rendering this difference not biologically relevant^37^. The differences registered between stearic and arachidic acid SLN are not in accordance with the literature^39^ that report similar biosafety among these fatty acids. Perhaps the longer chain of arachidic acid (when compared to stearic acid) interacts differently with the Tween^®^ 60 or directly with cells and causes less damage to the latter. Regardless, both SLN present a considerably low toxicity in fibroblasts, when used in concentrations up to 1 mg/mL, making them safe for use as biomaterials in feed according to the existing regulations, which states that an effect or condition is only considered toxic when it reduces viability by 30% or more^37^. These results indicate that the proposed SLN are not inherently toxic and could potentially be used in animals, however in vivo studies should still be performed to ensure their safety in actual animals.

### Differential scanning calorimetry assay

Studying the alterations that SLN undergo when subjected to different temperatures can provide very useful data, both to predict how they would react at high temperatures, such as those found in some industrial processes of feeds, and to characterize their stability over time. A DSC assay was performed to study the effects of high temperatures in the SLN and its results are presented in Table 4. The determined melting point for stearic and arachidic acids was 72.1 °C and 79.9 °C, respectively. These values were very similar when compared with the true values of 72.5 °C for stearic acid and slightly higher when compared with 75.5 °C for arachidic acid^40^, as expected since the components used were highly pure. Moreover, Lys appears to have no effect in any of the determined properties of the bulk materials, as expected considering its high melting point of 224.5 °C. In contrast, the SLN structure presented both a decreased melting point and onset values, as well as a high RI value.

**Table 4:** DSC results for nanoparticles composed of stearic (SLN S) or arachidic acid (SLN A) with or without lysine (Lys), as well as controls of stearic and arachidic acid and their physical mixtures with Lys. Values of ΔH (J/g), Onset (°C), Melting Point (°C) and recrystallization index (RI (%)) shown.

Sample	ΔH (J/g)	Onset (°C)	Melting point (°C)	RI (%)
SLN S without Lys	20.7	51.9	68.1	95
SLN S with Lys	21.3	59.1	68.4	97
SLN A without Lys	20.1	67.7	74.6	93
SLN A with Lys	19.6	64.7	74.0	91
Stearic acid	217.0	62.2	72.1	100
Stearic acid with Lys	219.1	59.5	71.4	100
Arachidic acid	217.0	68.0	79.9	100
Arachidic acid with Lys	215.0	68.7	78.0	100

The results obtained in the DSC analysis, regarding the melting points, are in accordance to the literature and can be explained by the presence of surfactants in SLN and due to their increased surface area to volume ratio^41^. The almost ten-fold decrease in the enthalpy (ΔH) values when comparing bulk materials to the same materials in SLN can also be attributed to the latter^41^. The SLN also presented a high RI that indicates a reduced chance of the particles undergoing polymorphism, in theory allowing reduced chances of Lys expulsion during aging^42^. It can also be seen that Lys, when considering materials in SLN, tends to slightly increase all the measured properties, probably due to interactions and alterations in the lipid matrix. These results, particularly the onset and melting point values, allows to state that the proposed SLN can safely be used in processes that reach temperatures of 60 °C without the risk of any degradation or damage, caused by temperature, to the SLN. This was expected since the chosen lipids were selected for their high melting point.

### Excipient grade components

A comparison of the effects of component purity, between the use of lab or excipient grade lipids and surfactants, was performed (Table 5). None of the assessed characteristics were significantly affected when the lab-grade lipid and surfactant were replaced with less pure components, except for the %Lys that slightly increased, when considering the sonication production method.

**Table 5:** Size (nm), polydispersity index (PdI), zeta potential (mV) and %Lys (%) values obtained for SLN produced using lab-grade and commercial lipids, n = 3.

	Lab-grade	Commercial	SEM	P value
Size (nm)	477	456	7	0.106
PdI	0.24	0.26	0.03	0.264
Zeta potential (mV)	− 42	− 35	4	0.111
%Lys (%)	61	64	1	0.041

*SEM* standard error of the mean.

The use of excipient grade components in SLN production does not influence their properties, enabling a reduction in the overall cost of the formulation without compromising its quality. Additionally, the use of these components slightly increases the %Lys, from 61 to 64%, probably since the less pure stearic acid used had a percentage of almost 40% of palmitic acid. The presence of these two solid lipids with different carbon lengths (C18 and C16, respectively) should create a less crystalline matrix with more imperfections where more Lys molecules can be trapped^43^.

### Production scalability

A comparison between the standard lab-scale sonication-based production and a more scalable method, based in HPH, was also performed^23^. The results show that both production methods can produce SLN with similar properties regarding their size, PdI and zeta potential, with no significant differences being found between the SLN (Table 6). Comparing the %Lys of both methods, they are also statistically similar regardless of the number of cycles.

**Table 6:** Size (nm), polydispersity index (PdI), zeta potential (mV) and %Lys (%) values obtained for nanoparticles (SLN) produced by the sonication method and by the high-pressure homogenization (HPH) method with 1 (HPH-1), 2 (HPH-2) and 3 (HPH-3) sequential cycles, n = 3.

Method	Sonication	HPH-1	HPH-2	HPH-3	SEM	P value
Size (nm)	456	364	415	430	8	0.087
PdI	0.26	0.30	0.25	0.24	0.01	0.364
Zeta (mV)	− 36	− 37	− 37	− 39	2	0.823
%Lys (%)	64	59	55	54	2	0.052

*SEM* standard error of the mean.

A tendency can be observed regarding the size of the SLN, where a reduced number of cycles seems to reduce it, whereas increasing this number seems to produce SLN with sizes more similar to the sonication-based production method. Despite no significant differences in the %Lys, a tendency can be observed as this value appears to decrease with increasing number of cycles. This decrease is probably due to the fact that when performing more than 1 cycle, the intermediate emulsions are placed at temperatures higher than the melting point between cycles^44^. The latter could cause the SLN to partly destabilize and release a portion of the encapsulated Lys, that is highly hydrophilic. Nevertheless, no significant differences exist, in any of the tested properties, when comparing the different production methods, indicating that it was possible to replicate the initial SLN, produced with a sonication-based method, using an HPH-based method. The latter is much simpler to scale up and thus enables a production on a much larger scale without compromising the SLN properties. Considering that the objective is to achieve an industrial production, the single cycle HPH method was selected for future applications, since fewer cycles translate into less time and steps required to produce the SLN. Additionally, a single cycle seemed to favor the Lys loading within the SLN.

### Nanoparticle lyophilization

The final SLN formulations are aqueous dispersions, which might not be the ideal form for a commercial product, both for preservation and transport reasons. To provide an alternative physical state for the SLN, an assay was performed to assess the viability of their possible lyophilization, rendering a dry and much lighter final product. The results obtained in this assay (Table 7) indicate that the lyophilization process does not significantly alter the tested properties of the SLN, except for the PdI.

**Table 7:** Comparison of sonication and high-pressure homogenization (HPH)-produced stearic acid solid lipid nanoparticles (SLN), before and after lyophilization (Lyo). Values shown for size, polydispersity index (PdI) and zeta potential, n = 3.

Formulation	Sonication SLN	Sonication SLN Lyo	SEM	P value
Size (nm)	562	452	28	0.098
PdI	0.23	0.29	0.01	0.011
Zeta potential (mV)	− 40	− 36	1	0.051

Formulation	HPH SLN	HPH SLN Lyo	SEM	P value
Size (nm)	352	394	16	0.166
PdI	0.30	0.27	0.01	0.049
Zeta potential (mV)	− 34	− 32	1	0.355

*SEM* standard error of the mean.

The alterations in PdI after SLN lyophilization, in both SLN, could be the result of mild aggregation or SLN erosion that widen, in the case of the sonication-produced SLN, or shorten the size distribution profile, in the case of the HPH-produced SLN. However, since the SLN size remains similar, the later most likely occurs on a very small scale and should not be very influential. The tendency observed in the size reduction of the sonication based SLN also supports this theory. Another tendency was found when comparing the zeta reduction (in modulus) of the sonication produced SLN, however the value is still high (in modulus) and should not influence the stability of the resuspended formulation. The obtained results indicated that the SLN could potentially be converted into a lyophilized form, with the inherent benefits, without compromising their efficiency. These findings should, however, be verified by performing more studies and assessing all SLN properties after the lyophilization process to determine if they retain all their original characteristics.

### Rumen stability assay

**Figure 2:**
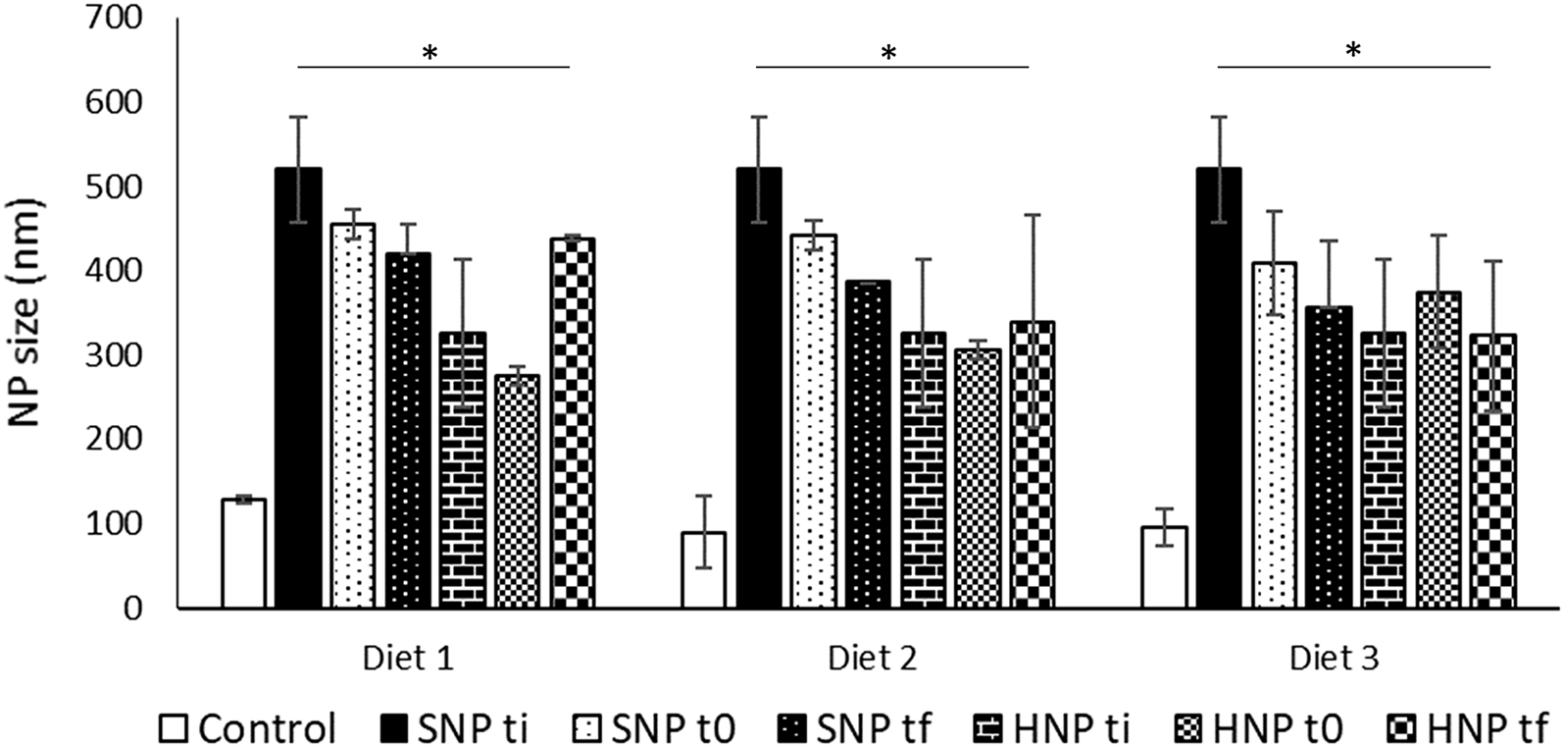
Particle size of solid lipid nanoparticle (SLN) produced by the standard method (SNP) and by high-pressure homogenization (HNP), where values are shown for the initial SLN (ti), for 0 h (t0) and 24 h (tf) of incubation in the rumen inoculum. The values are shown for SLN incubated in the rumen of cows fed with three different diets. Controls without SLN were used. * denotes statistically significant differences from the controls (P ≤ 0.05). No significant differences were found between SLN in either timepoint and the respective initial SLN (P ≤ 0.05).

To ensure that the ruminal resistance of SLN was not altered by a different composition and a different production method, both standard sonication- and HPH-produced SLN were subjected to a ruminal resistance assay, as previously described. This assay was performed using rumen inoculum collected from cows fed different diets to ensure true replicas (n = 3). No significant differences were found between SLN after production (*t*_*i*_), SLN with contact with the ruminal media (*t*_*0*_) and SLN after 24 h of incubation in the ruminal media (*t*_*f*_) for both production methods (Fig. 2).

The different production parameters considered, purity of components and SLN production method, were not expected to influence the ruminal digestion of the SLN as the lipid matrix, in theory, would not be greatly altered. However, it was still required to ensure that the introduced alterations did not interfere with the ruminal digestion of the SLN, since this property is essential. These results (Fig. 2) support the previous conclusions that SLN are indeed able to resist ruminal digestion, and prove that the newly proposed, less pure, HPH-produced SLN are also capable of resisting ruminal digestion. Moreover, all SLN at all timepoints were found to be significantly different from the controls (rumen inoculum without any SLN) indicating that the separation method was effective and that the results were not tainted by particles naturally present in the rumen.

### Shelf-life stability

**Figure 3:**
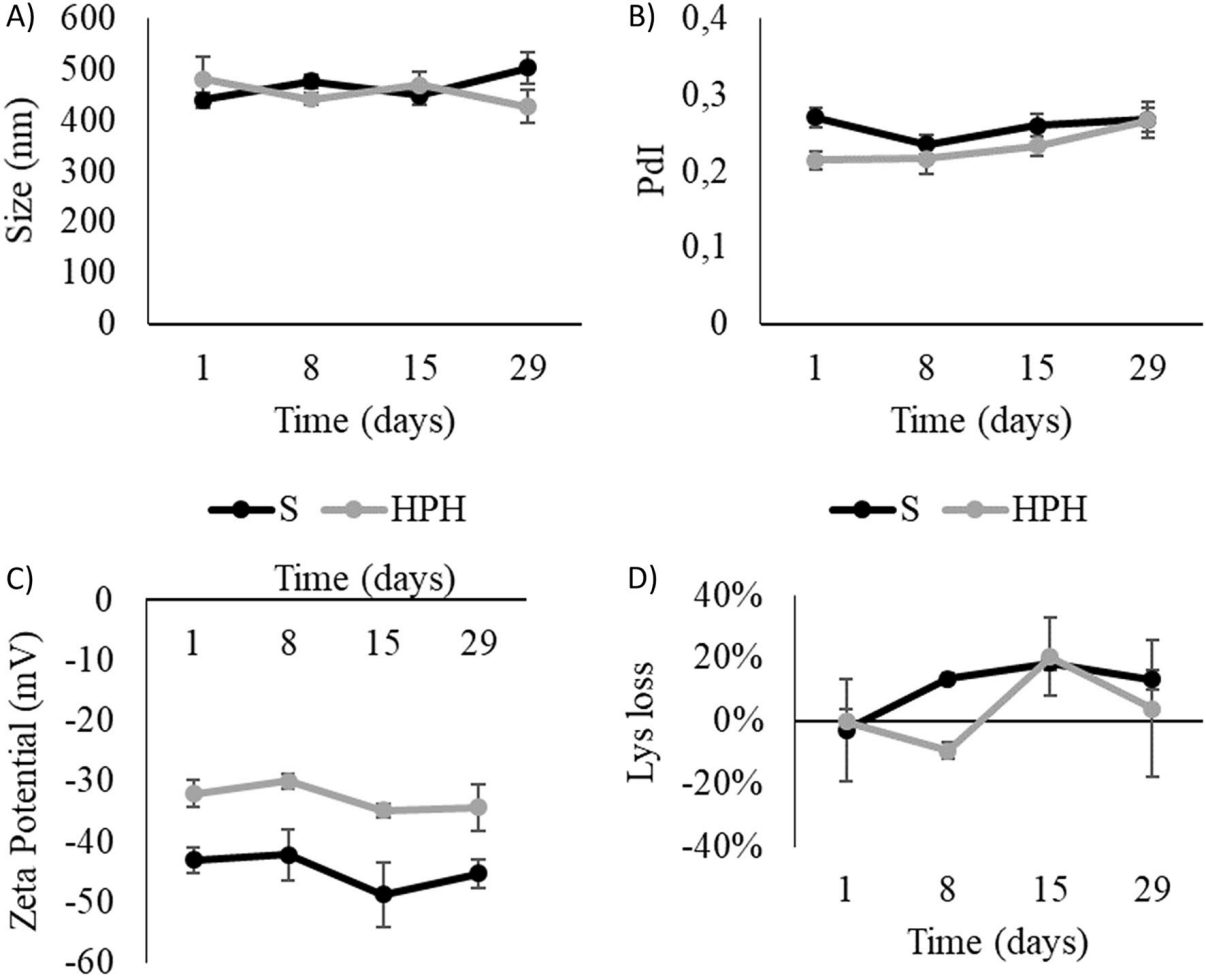
Size (**A**), polydispersity index (PdI) (**B**), zeta potential (**C**) and Lys losses (**D**) values for both nanoparticles produced by sonication method (S) and high-pressure homogenization (HPH) method over the period of 29 days of storage. No significant differences were found between day 1 of storage and any of the other tested timepoints (P ≤ 0.05).

When considering a product, its shelf-life, or the amount of time it can be stored while retaining its properties, is of extreme importance to assess its applicability. No significant differences were found in either standard sonication-produced or HPH-produced SLN for up to 29 days of storage (Fig. 3), indicating that the tested SLN could maintain their properties and remained viable for use at least within the tested time period. At this time only the stability of the SLN themselves was tested, but in order for these nanoparticles be used in animal feeding, future studies are needed to evaluate their stability namely when included in a total mixed ration (TMR).

### Industrial scale production

Considering that the results showed no significant changes in the SLN properties, regardless of the purity of the components or the production method, an attempt to produce SLN on a larger scale was performed. The latter was achieved using an industrial HPH system, currently used in the industry, to produce 3 L of SLN suspension at once. A comparison of these SLN with the SLN produced by the standard sonication method was performed and the results showed almost no significant changes in their properties, except for a bigger PdI and an increased %Lys (Table 8). The increase in size distribution profiles is due to, using this method, no pre-emulsion was created and the emulsion was formed by solely mixing the water with the lipids and directly placing it in the homogenizer. The latter is relevant because, in HPH methods, the qualities of pre-emulsion affect the properties of the final emulsion^45^. The increase in %Lys was also an unexpected improvement, as it would increase the amount of Lys that the SLN are able to transport. This is probably justified by the larger size of the SLN that possibly can accommodate more Lys within their matrix. It should also be noted that larger volumes are more difficult to properly homogenize than smaller ones and the volume used in the larger HPH system was over 300-fold larger than the volume used in the smaller HPH, which could also help to explain the differences between the SLN produced with both HPH systems.

**Table 8:** Size (nm), polydispersity index (PdI), zeta potential (mV) and %Lys (%) values obtained for SLN of stearic acid with Tween^®^ 60 produced using the standard sonication method and the larger scale high-pressure homogenization (HPH) method, n = 3.

	Standard	HPH	SEM	P value
Size (nm)	456	950	138	0.054
PdI	0.26	0.34	0.01	0.005
Zeta potential (mV)	− 36	− 40	1	0.172
%Lys	64	70	1	0.022

*SEM* standard error of the mean.

## Conclusions

The goal of this work was to determine if the previously developed rumen resistance Lys-delivery systems were viable candidates for animal supplementation, using in vitro and ex vivo approaches. Both SLN of stearic and arachidic acids were found to resist digestion in the abomasum and in the small intestine. The proposed SLN also appear to be slowly degraded in the blood, thus releasing their Lys content, particularly stearic acid SLN that were completely destroyed after 24 h of incubation. Moreover, the in vitro assays performed showed no inherent toxicity for both stearic or arachidic acid SLN, within the concentrations to be used in vivo, and could therefore be considered for applications in living animals. However, additional studies should be performed to properly determine release profile and kinetics overtime, which could help to further improve the nitrogen-usage in the animal by increasing the Lys-usage efficiency. The results showed that SLN produced by HPH possessed similar properties to those produced by the standard sonication-based method in terms of size, PdI, zeta potential and %Lys without compromising their ability to resist ruminal digestion. The DSC results also enable us to conclude that the SLN can resist additional production processes under temperatures of 60 °C, whereas the lyophilization study indicated that they could be used in a powder form rather than that of liquid suspension. These SLN were also able to retain their physical properties for up to a month of storage at RT. To conclude, the tested SLN possess important qualities when considering an industrial application and should be considered promising alternatives to the current rumen-protected products. However, it should be noted that this work was primarily performed in vitro and in vivo follow-up studies should be conducted to confirm and validate the obtained results.

## Materials and methods

### Materials

Lab grade stearic acid was purchased from Merck (© Merck KGaA, Darmstadt, Germany) and excipient grade stearic and Tween^®^ 60 were obtained from Acofarma (^®^ Acofarma–Acofarma Distribución, S.A. BCN, Spain). Arachidic acid, lab grade Tween^®^ 60, l-lysine monohydrochloride, lithium carbonate, dansyl chloride, ethylenediaminetetraacetic (EDTA) acid disodium salt dihydrate, dimethyl sulfoxide (DMSO), 3-(4,5-dimethylthiazol-2-yl)-2,5-diphenyltetrazolium bromide (MTT), Triton^tm^ X-100, methylamine hydrochloride, triethylamine, sodium acetate, pepsin and pancreatin (from porcine pancreas) were purchased from Sigma-Aldrich (St. Luis, MO, USA), l-phenylalanine ethyl-ester hydrochloride from Fluka (Fluka Chemie GmbH, Buchs, Switzerland), acetic acid from VWR Chemicals (VWR International S.A.S., Fontenay-sous-Bois, France), acetonitrile and methanol from Honeywell (Honeywell Riedel-de Häen AG, Seelze, Germany). Dulbecco’s Modified Eagle’s Medium (DMEM), penicillin–streptomycin (10,000 U/mL) mix, fetal bovine serum (FBS) and amphotericin B (Fungizone) were purchased from Gibco^®^ (Invitrogen Corporation, UK). FaSSGF and FeSSIF were purchased from Biorelevant.com Ltd (London, UK). Aqueous solutions were prepared with double-deionized water (Arium Pro, Sartorius AG, Göttingen, Germany).

### Methods

#### Sonication-based production method

An organic-solvent free method based on ultrasounds, using arachidic or stearic acid as solid lipid and Tween^®^ 60 as surfactant, was used to produce SLN^17^. In short, lipid and surfactant were melted at 85 °C in a water bath. A solution of Lys was prepared using ultra-pure water, heated at the same temperature and 4.4 mL of this solution added to the melted lipids. The resulting emulsion was sonicated using a probe-type sonicator for 5 min at 80% amplitude (model VCX-130 with a VC 18 probe, Sonic & Materials Inc., Newtown, CT, USA) to form a nanoemulsion and afterwards allowed to cool down at room temperature (RT), rendering an aqueous suspension of SLN, which was stored at RT until further use.

#### High-pressure homogenization production method

An organic-solvent free method based on HPH^45^, using stearic acid as solid lipid and Tween^®^ 60 as surfactant, was used to produce SLN. In short, 1600 mg of lipid and 260 mg of surfactant were melted at 80 °C in a water bath. A solution of Lys at 6.6 mg/mL was prepared using ultra-pure water and heated at the same temperature. The melted lipids were mixed with 60 mL of Lys solution, at the same temperature, to form an emulsion. This emulsion was pre-homogenized, for 5 min at 12,000 rpm (T25 digital, ULTRA TURRAX^®^-IKA^®^-Werke GmbH & Co. KG, Staufen, Germany), and then kept at 80 °C in a water bath until further processing. Using a 10 mL syringe, 9 mL of the previous emulsion were placed in the HPH chamber (SPCH-10-EP Ultra High-Pressure Homogenizer, Stansted Fluid Power Products Ltd, Stansted, UK) and homogenized at 5.5 Bar and 80 °C. The resulting nanoemulsions were collected and either placed in the homogenizer for re-processing (additional cycle) or allowed to cool at RT to enable SLN formation. After the adequate HPH cycles were performed, the SLN dispersion were placed in glass vials and stored at RT until further usage. Three different number of cycles were tested: 1 cycle, where the resulting emulsion was stored after the first cycle; 2 cycles, where the resulting emulsion was subjected to another HPH cycle; and 3 cycles, where the resulting emulsion was subjected to 2 consecutives additional HPH cycles.

#### Industrial high-pressure homogenization production method

An organic-solvent free method based on HPH^45^, using stearic acid as solid lipid and Tween^®^ 60 as surfactant, was used to produce SLN similarly to the previous method. In short, 275 g of lipid and 65 g of surfactant were melted at 80 °C in a water bath. A 3 L solution of Lys at 6.6 g/L was prepared using deionized water and heated at the same temperature. The latter solution was added to the melted lipids and the resulting emulsion homogenized (homogenizer Rannie™ model Bluetop, Copenhagen, Denmark) at 200 Bar. The resulting nanoemulsion was stored at RT until further usage.

#### Nanoparticle diameter and zeta potential

The SLN formulations were characterized in terms of mean diameter and size distribution profile (polydispersity index—PdI) by dynamic light scattering (DLS) using a 90Plus Particle Size Analyzer (Brookhaven Instruments Corporation, Holtsville, NY, USA) and in terms of zeta potential by electrophoretic light scattering using a ZetaPALS Zeta Potential Analyzer (Brookhaven Instruments Corporation, Holtsville, NY, USA). A wavelength of 660 nm, a temperature of 20 °C, and a detection angle of 90° were used in the measurements and the refractive indexes were 1.59 and 1.33, for the SLN and the solvent (water), respectively^17^. All samples were diluted using ultra-pure water.

#### Lysine quantification

Lysine encapsulation efficiency (%Lys) was determined as the difference between the total amount of Lys added in the preparation of SLN and the amount of Lys outside the SLN (Eq. 1). The percentage of Lys in the SLN composition, Lys loading capacity (%LC), was also calculated (Eq. 2).

1 $$\mathrm{\%Lys }\left(\mathrm{\%}\right)= \frac{\mathrm{Total \; amount \;of \;Lys }-\mathrm{ amount \; of \; Lys \; in \; the \;supernatant}}{\mathrm{Total \; amount \; of\; Lys}} \times 100$$

2 $$\mathrm{\%LC }\left(\mathrm{\%}\right)= \frac{\mathrm{Amount \; of \; encapsulated \; Lys}}{\mathrm{Amount \; of \; Lipid}+\mathrm{Amount \; of \;Surfactant}} \times 100$$

Firstly, the SLN suspensions were diluted 100× and filtrated with Amicon^®^ Ultra Centrifugal Filters Ultracell-50 kDa (EMD Millipore, Darmstadt, Germany) at 2200×*g* for 33 min using an Allegra^®^ X-15R centrifuge (Beckman Coulter, Pasadena, CA, USA). The supernatant was then collected for quantification using a previously developed, high-performance liquid chromatography-based method for the purpose of quantifying Lys, described elsewhere^46^.

#### Nanoparticle stability in abomasal conditions

The abomasal medium was mimicked using fasted state simulated gastric fluid (FaSSGF)^47^, prepared according to manufacturer specifications, with a pH of 1.6, supplemented with pepsin at 1 mg/mL. Both stearic and arachidic acid SLN were incubated, in triplicate, in the previously mentioned medium, at 39 °C, under light stirring^48^. The same medium without any SLN was also incubated and used as control, also in triplicate. Aliquots were collected from each replica at 0 h (*t*_0_) and at 1 h (*t*_f_) of incubation^49,50^ for further analysis. Both samples and controls were assessed in terms of size using DLS and compared with the values obtained for SLN without any contact with the abomasal mimicking medium (*t*_i_).

#### Nanoparticle stability in intestinal conditions

The intestinal medium was mimicked using fed state simulated intestinal fluid (FeSSIF)^47^, prepared according to manufacturer specifications, with a pH of 5, supplemented with pancreatin at 3 mg/mL. Both stearic and arachidic acid SLN were incubated, in triplicate, in the previously mentioned medium, at 39 °C, under light stirring^48^. The same medium without any SLN was also incubated and used as control, also in triplicate. Aliquots were collected from each replica at 0 h (*t*_0_) and at 2 h (*t*_f_) of incubation^50^, for further analysis. Both samples and controls were assessed in terms of size using DLS and compared with the values obtained for SLN without any contact with the intestinal mimicking medium (*t*_i_).

#### Nanoparticle stability in the bloodstream

The SLN stability in the bloodstream was assessed using fresh bovine blood. Bovine blood was collected from the jugular vein of Holstein Friesian cattle during their slaughter at a commercial abattoir (PEC Nordeste—Indústria de Produtos Pecuários do Norte, Penafiel, Portugal). Blood was harvested and stored in 0.5 L Schott flasks, previously coated with EDTA. The collection and use of this animal byproduct was authorized by the Portuguese Directorate-General of Food and Veterinary Medicine of the Ministry for Agriculture and Sea (authorization number: N.12.006.UDER). Both stearic and arachidic acid SLN were incubated in this medium, in triplicate, at 39 °C, under light stirring, protected from the light and sealed to avoid oxidation (only in contact with the atmosphere during aliquot collection). The same medium without any SLN was also incubated, in triplicate, and used as a negative control. Aliquots were collected at 0, 3, 6, 9, 12 and 24 h of incubation. Both samples and controls were centrifuged at 2000×*g* for 15 min to separate blood components, and the supernatants collected for further analysis. All samples and controls were assessed in terms of size using DLS and compared with the values obtained for SLN without any contact with any of the medium (*t*_i_).

#### Rumen resistance assay

Ruminal contents were obtained from three multiparous (number of parity = 2) adult Holstein cows, dry and not pregnant that were fitted with a ruminal cannula (10 cm diameter; Bar Diamond Inc., Parma, ID, previously approved and licensed by the Portuguese Directorate-General of Food and Veterinary Medicine of the Ministry for Agriculture and Sea, permit 0421/000/000/2015). The cows were housed at the Vairão Agricultural Campus of School of Medicine and Biomedical Sciences, University of Porto (Vila do Conde, Portugal). Cows were handled in strict accordance with good animal practices as defined by the national authority and European Union Directive 2010/63/EU. Experimental animal procedures were approved by the Local Animal Ethics Committee of School of Medicine and Biomedical Sciences, University of Porto, licensed by the Portuguese Directorate-General of Food and Veterinary Medicine of the Ministry for Agriculture and Sea, and conducted by trained scientists (FELASA category C). This study was also performed in accordance with ARRIVE guidelines. Each cow was fed a TMR, based on corn silage or haylage, and always had fresh drinking water available. Ruminal contents were collected before the morning meal from the 4 quadrants of the rumen and placed in a 4 L pre-warmed (39 °C) thermal jug. In the laboratory, the ruminal digesta of each cow was homogenized and strained through 4 layers of linen cloth at 39 °C in O_2_-free CO_2_ atmosphere. The interval between the collection of the ruminal contents and incubation never exceeded 60 min. One part of the strained ruminal fluid, pH 6.3, was diluted anaerobically into 4 parts of Kansas State Buffer^51^ and mixed at 39 °C in an O_2_-free CO_2_ atmosphere. Thirty milliliters (25 mL of buffered ruminal fluid and 5 mL of each SLN formulation) were dispensed anaerobically into 125 mL serum bottles (Sigma-Aldrich Inc., St. Louis, MO, USA) containing 250 mg (dry matter) of wheat straw, sealed with butyl rubber stoppers and aluminum crimp caps (Sigma-Aldrich Inc., St. Louis, MO, USA), and incubated in a water bath at 39 °C. Each SLN formulation was added to previously prepared serum bottles and incubated in quadruplicate. Blanks, containing no SLN, were also incubated in quadruplicate, to be used as controls. Two of these replicates of each sample and blanks were placed in an ice bath immediately after the addition of the SLN formulation to serve as negative controls (*t*_*0*_ samples, where *t* represents the time and _*0*_ indicates that no incubation occurred). Fermentations were stopped after 24 h by cooling the bottles in an ice-slurry bath (*t*_*f*_ samples, where *t* represents the time and _*f*_ indicates that the incubation occurred until the end of the designated time period). The samples were subsequently compared with SLN that did not contact the rumen inoculum (*t*_*i*_ samples, where *t* represents time and _*i*_ represents the SLN characterized after production)^17^.

The contents of the flasks were transferred to Falcon tubes and centrifuged at 500×*g* for 5 min to separate any remaining feed and the heaviest microorganisms, such as protozoa, from the SLN. The supernatant was collected and centrifuged at 30,000×*g* for 20 min to separate the SLN from the bacteria and other microorganisms that did not deposit in the first centrifugation. Both supernatants and deposits were collected for characterization in terms of size, PdI and zeta potential. This procedure was also performed on rumen inoculum and buffer mixture without the addition of any SLN (blanks), and these results were used as controls to verify the separation of the SLN and rumen inoculum^17^.

#### Cell culture studies

The L929 mouse fibroblastic cell line was selected for the in vitro biosafety characterization of the SLN, as they are recommended for biomaterial safety evaluation for biomedical devices and food applications^37^.

Cells were cultivated in DMEM, supplemented with 10% (v/v) FBS, 1% (v/v) penicillin/streptomycin mixture and 1% (v/v) amphotericin B, at 37 °C in 5% CO_2_ atmosphere. The medium was replaced every 3 days and when cells were confluent, they were detached from the culture flasks with a scrapper (Nunc™ Cell Scrappers, Thermofisher Scientific, Waltham, MA USA). After this physical detachment, cells were centrifuged at 300×*g* for 5 min in a Heraeus™ Multifuge™ X1R centrifuge (Thermo Fisher Scientific; Waltham, USA) and then resuspended in fresh DMEM. Counting of viable cells was performed using a Neubauer chamber (Improved Neubauer Bright-Line, Boeco; Germany) in a Motic^®^ AE2000 Binocular Inverted Microscope (Motic Electric Group Co., Ltd; Xiamen, Fujian, China)^52^.

To assess the impacts of SLN in cellular viability, a MTT assay was performed^53^. Cells were firstly collected and seeded in 96-well plates (5 × 10^4^ cells per well) with 100 µL of fresh culture medium. After cellular adhesion, the medium was discarded and replaced by fresh DMEM medium, supplemented with SLN or free Lys. The SLN were added to achieve the final concentrations of 0.125, 0.25, 0.5, 1 and 2 mg of SLN per mL of medium, and free Lys was added to achieve the same concentrations that would be found in the respective mass of SLN. Cells with no SLN nor free Lys were included to be used as positive controls and cells incubated with 1% triton X-100 were used as negative controls. The cells were incubated, in quadruplicate, for all the aforementioned conditions for 24 h, at 37 °C in 5% CO_2._ After incubation, the medium was completely discarded and replaced by 100 µL of a 0.5 mg/mL MTT solution. The plate was incubated for 2 h, at 37 °C in 5% CO_2,_ after which the MTT solution was discarded and 200 µL of DMSO were added to solubilize the formazan crystals formed inside the cells. Finally, the absorbance was read at 570 and 630 nm using a Synergy™ HT Multi-Mode Microplate Reader (Biotek Instruments, Winooski, VT, USA). The absorbance at 630 nm was subtracted from the absorbance at 570 nm to remove interferences from the medium, and the cellular viability was calculated by comparing the treated cells with the control cells. The results were normalized and compared with the positive controls (in theory, 100% of viability).

#### Differential scanning calorimetry

The crystallinity degree and lipid polymorphism of the SLN were evaluated by differential scanning calorimetry (DSC) using a PerkinElmer Pyris 1 DSC 200 F3 Maia^®^ (Netzschm, MA, USA). Samples were weighed (2–5 mg) directly on aluminum pans and an empty pan was used as a reference. All samples were scanned between 20 °C and 90 °C, with a heat rate of 10 °C per min and cooled down at 40 °C per min. This analysis was performed in the SLN, both loaded and not loaded with Lys, the bulk materials that constitute them and mixtures of these materials with Lys. The values of onset (°C), melting point (°C) and enthalpy (ΔH) were calculated from the data using OriginPro (OriginLab), whereas the recrystallization index (RI) was calculated using the Eq. (3)^41^.

3 $${\text{RI}}\left( \% \right) = \frac{{\Delta {\text{H}}\;{\text{SLN}}\;\left( {{\text{J}}/{\text{g}}} \right)}}{{\Delta {\text{H}}\;{\text{bulk}}\;{\text{material}}\left( {{\text{J}}/{\text{g}}} \right) \times {\text{Concentration}}\;{\text{lipid}}\;{\text{phase}}}} \times 100$$

#### Lyophilization assay

Firstly, 2 mL of SLN dispersion, supplemented with 1% AEROSIL^®^, were frozen overnight at − 80 °C (Deep Freezer GFL^®^, Burgwedel, Germany) and were afterwards lyophilized using a laboratory freeze drier (LyoQuest-85 plus v.407, Telstar^®^ Life Science Solutions, Terrassa, Spain) for 72 h at − 80 °C under a pressure of 0.4 mbar. The lyophilized SLN were resuspended in the same volume of water (2 mL) and characterized in terms of size, PdI and zeta potential as previously described.

#### Shelf-life stability assay

To evaluate the shelf-life of the developed SLN formulations, they were stored at RT and assessed, after 1, 2 and 4 wks, in terms of their size, size distribution profile, zeta potential and %Lys, using the previously described methods.

#### Statistical analysis

All statistical analyses were performed with IBM SPSS Statistics (SPSS 27.0, Armonk, NY, USA). Univariate one-way analysis of variance (ANOVA) was performed to compare multiple groups of independent samples. When the effect was statistically significant (P ≤ 0.05), the differences between the respective groups were compared with a post-hoc test (Pairwise, P ≤ 0.05).

## Data Availability

The datasets used and/or analysed during the current study are available from the corresponding author on reasonable request.
