# Author Correction: Production of rumen- and gastrointestinal-resistant nanoparticles to deliver lysine to dairy cows

**DOI:** 10.1038/s41598-023-46598-8

**Published:** 2023-11-07

**Authors:** João Albuquerque, Ana R. Neves, Ingrid Van Dorpe, António J. M. Fonseca, Ana R. J. Cabrita, Salette Reis

**Affiliations:** 1 LAQV, REQUIMTE, Department of Chemical Sciences, FFUP, Rua Jorge Viterbo Ferreira n.º 228, 4050‑313 Porto, Portugal; 2 School of Medicine and Biomedical Sciences (ICBAS), University of Porto, Rua Jorge Viterbo Ferreira n.º 228, 4050‑313 Porto, Portugal; 3 CQM+‑Centro de Química da Madeira, Universidade da Madeira, Campus da Penteada, 9020‑105 Funchal, Portugal; 4 PREMIX-Especialidades Agrícolas e Pecuárias, Lda, Parque Indústrial II–Neiva, 4935‑232 Viana do Castelo, Portugal; 5 LAQV, REQUIMTE, School of Medicine and Biomedical Sciences (ICBAS), University of Porto, Rua Jorge Viterbo Ferreira n.º 228, 4050‑313 Porto, Portugal

Correction to: *Scientific Reports* http://doi.org/10.1038/s41598-023-43865-6, published online 04 October 2023

The original version of this Article contained an error in the Acknowledgements section.

“This work received financial support from FCT (Fundação para a Ciência e a Tecnologia) and FEDER funds under Program PT2020 (PT2020UID/QUI/50006/2020).”

now reads:

“This work received financial support from Ministério para a Ciência e Tecnologia – FCT Fundação para a Ciência e a Tecnologia through national funds to the project UIDP/50006/2020.”

The original Article has been corrected.

